# Association between TYMS Expression and Efficacy of Pemetrexed–Based Chemotherapy in Advanced Non-Small Cell Lung Cancer: A Meta-Analysis

**DOI:** 10.1371/journal.pone.0074284

**Published:** 2013-09-10

**Authors:** Ting Wang, Chang Chuan Pan, Jing Rui Yu, Yu Long, Xiao Hong Cai, Xu De Yin, Li Qiong Hao, Li Li Luo

**Affiliations:** 1 Department of Medical Oncology, Sichuan Cancer Hospital & Institute, Chengdu, Sichuan, PR China; 2 Department of Emergency, West China Second University Hospital, Sichuan University, Chengdu, Sichuan, PR China; Sudbury Regional Hospital, Canada

## Abstract

**Background:**

The predictive value of thymidylate synthase (TYMS) to sensitivity to pemetrexed-based chemotherapy in advanced non-small cell lung cancer (NSCLC) patients is controversial. We conducted a meta-analysis of all relevant published data to assess the association of TYMS expression with the clinical outcomes of pemetrexed-based regimen in advanced NSCLC.

**Patients and Methods:**

We conducted an electronic search using using PubMed, Embase, OVID and Cochrane Library databases and manual search. Pooled odds ratio (OR) for the response rate and hazard ratio (HR) for the overall survival and progression free survival were calculated using the software Revman 5.0.

**Results:**

There were 11 studies (*n*=798) met our criteria for evaluation. Response rate to pemetrexed-based regimen was significantly higher in patients with low/negative TYMS (OR=2.96, 95%CI [1.81, 4.86] *P*<0.0001). Patients with low/negative TYMS who were treated with pemetrexed-based regimen had longer progression free survival (HR 0.50, 95%CI [0.41, 0.61] *P* <0.00001) and overall survival (HR 0.41, 95%CI [0.22, 0.78] *P*=0.007) than those with high/positive TYMS.

**Conclusions:**

Low/negative TYMS expression was significantly associated with higher response rate, longer median survival and longer progression free survival for advanced NSCLC patients receiving pemtrexed-based chemotherapy. Hence, TYMS may be a potential predictor of sensitivity to pemtrexed-based chemotherapy in advanced NSCLC. Large scale prospective clinical trials are still warranted.

## Introduction

Lung cancer has been estimated as the most common cancer in the world for several decades [1–7]. An estimated 1.61 million people across the world were diagnosed with lung cancer which accounts for an estimated 1,378,400 deaths world-wide in 2008 [1]. Approximately 85% of all cases are non-small-cell lung cancer (NSCLC) at diagnosis and only 15% of lung cancers are detected at the localized stage [8]. Platinum-based doublet combination chemotherapy is regarded as the standard first-line treatment for advanced NSCLC that usually consists of a platinum compound with a third-generation agent (paclitaxel, docetaxel, pemetrexed, or vinorebine) [9,10].

Pemetrexed, a multitargeted antifolate cytotoxic chemotherapy agent, which inhibits at least three target enzymes in the folate pathway (thymidylate synthase, dihydrofolate reductase, and glycinamide ribonucleotide formyl transferase), is approved as standard second-line treatment for advanced NSCLC [11,12]. These enzymes may serve as biomarkers for predicting treatment efficacy of pemetrexed. A recent in vitro study mentioned that down-regulation of thymidylate synthase(TYMS) gene was found in pemetrexed-sensitive lung cancer cell lines [13]. Recent studies have reported that TYMS expression of tumor tissues was significantly related to the prognosis in patients with several malignant tumors such as mesothelioma, gastric cancer and colorectal cancer [14–18].

For NSCLC patients, TYMS expression has been studied to predict the survival of patients with resectable NSCLC [19,20]. Several recent studies reported that low TYMS expression was associated with better response and/or survival when treated with pemtrexed-based regimens in NSCLC patients [21–24]. But some other studies didn’t show the significant association between TYMS expression and efficacy of pemtredxed-based chemotherapy in NSCLC [25,26]. However, the association between TYMS expression and treatment efficacy of pemetrexed in NSCLC is unclear. Considering the conflicting results of these studies, a meta-analysis is performed to evaluate whether thymidylate synthase (TYMS) is a predictive biomarker of efficacy of pemetrexed-based regimen in advanced NSCLC and provide more persuasive evidence for our clinical practice.

## Methods and Patients

We searched and analyzed data of the published case-control and cohort studies in which sensitiveness to pemetrexed was compared between TYMS high/positive and TYMS low/negative patients. The pooled odds ratio (OR) for the response rate and hazard ratio (HR) for median survival and progression free survival and their 95% confidence interval (CI) were calculated.

### Study Inclusion Criteria and Exclusion Criteria

Only published studies were included regardless of publishing date and study design. Publishing language was restricted to English. The study subjects should be patients with pathologically proven advanced NSCLC received pemetrxed-containing regimens. TYMS expression should be detected with immunohistochemistry (IHC) or real-time reverse transcriptase PCR (RT-PCR). Study that didn’t provide at least one of outcomes objective response rate, median survival or survival time will be excluded.

### Types of Participants

The meta-analysis included patients who were diagnosed advanced NSCLC with stage IIIA, IIIB or IV. Eligible patients for the study were ≥ 18 years old and had histologically or cytologically confirmed advanced NSCLC suitable for chemotherapy. The relapse patients will also be included. Both the treatment-naive patients and those who received previous treatment (such as surgery, radiotherapy, target therapy or chemotherapy) will be included. Patients who received concurrent radiotherapy are eligible for inclusion.

### Search Strategy

Two investigators (T Wang, JR Yu) searched the articles independently according to the inclusion criteria mentioned above. Electronic search was conducted in the database PubMed, Embase, OVID and Cochrane Library. We searched the articles published from inception to May 2013. The search terms were (thymidylate synthase OR TYMS OR TS) and (non-small cell lung cancer OR non-small cell lung carcinoma) and pemetrexed and chemotherapy, and any combination of key words were used to electronic search. The manual search was applied in the reference of included studies. We only searched the articles published in English.

### Quality Assessment

Quality of the studies was assessed using the Newcastle-Ottawa Quality Assessment Scale for cohort studies. This scale is an eight-item instrument that allows for assessment of patient population and selection, study comparability, follow-up, and outcome of interest. Interpretation of the scale is performed by awarding points, or stars’, for high-quality elements. Stars are then added up and used to compare study quality in a quantitative manner, which was recommended by the Cochrane Non-Randomized Studies Methods Working Group [27]. Studies with 5 or more stars are defined as high quality studies and will be included. Quality assessment was performed by two investigators (T Wang and LL Luo) independently. Data were adjudicated by 2 additional investigators (JR Yu and XH Cai) according to the original articles after data extraction and assessment. Any disagreement will be present to discuss within all authors.

### Data Extraction

A standard data extraction form was used. Two investigators (T Wang, JR Yu) extract the information from each study independently. Any dispute was solved via discussion. Only if both investigators approved the study that it could be included in this meta-analysis. Characteristics of the study including author name, publication time, ethnicity, study design, sample size, age, disease stage, ECOG PS, TYMS detection method and outcomes (response to pemetrexed, overall survival, progression free survival and hazard ratio) were all recorded. When there was overlapped data between studies, we will included the study reports the largest amount of patients and exclude the others. If original hazard ratio was not reported the survival curves of overall survival and time to progression will be extracted to calculate hazard ratio according to the methods described by Tierney in 2007 [28].

### Statistical Analysis

The primary end points were objective response rate, progression free survival, and overall survival. The association between TYMS and response rate was expressed as odds ratio (OR). The association between TYMS and PFS or OS was expressed as a hazard ratio (HR). Statistical heterogeneity between studies was examined using both the Cochrane Q statistic (significant at P<0.1) and the I^2^ value. I^2^>50% were considered to represent significant heterogeneity. A fixed-effect model was used when heterogeneity was not detected (*P*>0.10); otherwise, a random-effect model was used. All statistical analysis was performed by Review manager 5.0 (http://www.cochrane.org). The pooled OR and its 95% confidence intervals (CIs) were calculated using Mantel–Haenszel formula (fixed-effect model) or Dersimonian–Laird formula (random-effect model). For quantitative evaluation of PFS and OS results, HR was used to estimate the impact of TYMS expression on PFS and OS of patients received pemetrexed-based chemotherapy. HR, variance, 95% CI, log(HR) and se(log(HR)) for each study were extracted or calculated based on the published studies according to the methods described by Tierney in 2007 [28]. Kaplan-Meier curves were read by Engauge Digitizer version 4.1 (http://digitizer.sourceforge.net/). A significant two-way P value for comparison was defined as *P*<0.05. The results were described by forest plots, every square represents each study’s OR or HR estimate. The pooled OR or HR is symbolized by a solid diamond at the bottom of the forest plot and the width of the square represents the 95% CI of OR or HR. The size of the square represents the weight that the corresponding study exerts in the meta-analysis. Subgroup analysis was performed to explore the influence of ethnicity and detecting method in the outcomes. Publication bias was evaluated using the funnel plot and the Begg’s test by Stata 11.0.

## Results

### Search Results

**Figure 1:**
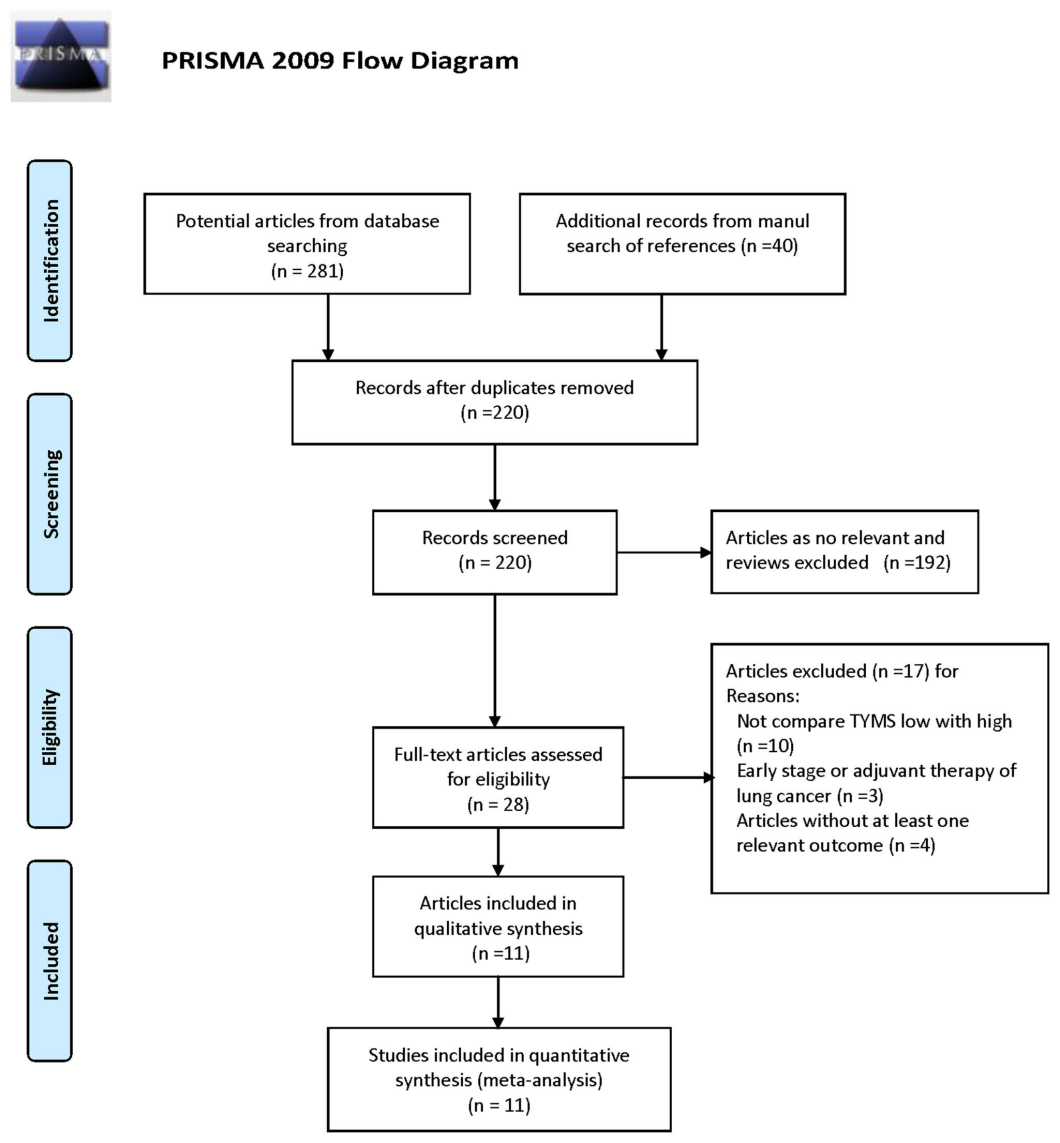
PRISMA Flow chart of the search result of the meta-analysis.

We identified 281 potentially relevant studies from electronic database search and 40 studies from manual search of references. 101 publications were excluded because duplication. 192 irrelevant studies and reviews were excluded. Afterwards, 28 articles were read in full independently by two investigators (T Wang, JR Yu). Finally, 11 studies of 798 patients were included in the final analysis [21–26,29–33]. Figure 1 shows the flowchart of the search results. The following data including year of publication, number of patients, ethnicity, TYMS detecting method, disease stage, performance status, age, response rate (RR), OS and PFS were extracted from each study. Most of included studies were retrospective studies and a few were prospective cohort studies. Characteristics of the eligible studies are listed in Table 1. Nine of eleven studies were performed in Asians and two in Caucasians. Nine studies utilized IHC and three utilized RT-PCR to detect the TYMS expression. All studies included stage III/IV patients, while only one study included 16 relapsed and stage IIIA patients which was included in the analysis. Ten except one studies reported the treatment line of pemetrexed-based regimens which was from 1^st^ line therapy to 4^th^ line therapy. Most studies included pre-treated patients. The treatment regimens varied a lot, including pemetrexed single agent, pemetrexed plus platinum, chemotherapy plus concurrent radiotherapy, and chemotherapy followed by radiotherapy. Six studies reported the median cycles of pemetrexed-based chemotherapy, and most of the patients received at least 4 cycles of pemetrexed-based therapy. As reported, the ECOG performance status ranged from 0 to 3. Four studies didn’t report the performance status.

**Table 1:** Characteristics of studies included in the meta-analysis.

Study(year)	Ethnicity	Patient (N)	TYMS detecting method	Disease stage	Treatment regimen	Treatment line	Chemotherapy cycle (range)	ECOG PS	Median age (year)	Evaluable for response (n)	TYMS low/negative		TYMS high/positive		Quality score	Risk of bias
											RR(n)	Total(n)	RR(n)	Total(n)		
Chang MH 2010 [25]	Asian	110	IHC	IIIb/IV	Pemetrexed	1^st^, 2^nd^, 3^rd^, 4t^h^	4 (1-22)	0-3	59	52	NR	NR	NR	NR	6 star	different detecting method, different therapy line, only Asian
Chen CY 2011 [21]	Asian	42	IHC	IIIb/IV	Pemetrexed + Radiotherapy	2^nd^, 3^rd^, 4^th^	NR	NR	61.5	42	5	22	3	20	6 star	different detecting method, different therapy line, only Asian, not report PS
Gadgeel SM 2011 [24]	Caucasian	28	IHC	IIIa/IIIb	Pemetrexed + cisplatin + Radiotherapy followed by docetaxel	NR	3+3	0-1	60	16	NR	NR	NR	NR	7 star	different detecting method, different therapy line, not report PS, only Caucasian,
Igawa S 2012 [23]	Asian	104	IHC	IIIb/IV	Pemetrexed	Pre-treated	4 (1-15)	0-3	65	54	5	31	0	23	6 star	different detecting method, different therapy line, only Asian
Lee SH 2013 [31]	Asian	41	IHC	IIIb/IV	Pemetrexed + cisplatin	1^st^	4 (1-9)	NR	68	NR	NR	NR	NR	NR	6 star	different detecting method, different therapy line, only Asian, not report PS
Nicolson MC 2013 [32]	Caucasian	70	IHC/RT-PCR	IIIb/IV	Pemetrexed + cisplatin	1^st^	4	0-1	65.1	60/61	15/14	36/39	3/2	24	6 star	different detecting method, different therapy line, only Caucasian
Park CK 2009 [33]	Asian	98	IHC	relapse/III/IV/	Pemetrexed	2^nd^, 3^rd^ ,4^th^	NR	NR	62	98	5	39	5	59	5 star	different detecting method, different therapy line, only Asian, not report PS
Sun JM 2011 [22]	Asian	193	IHC	IIIb/IV	Pemetrexed/Pemetrexed + platinum	1^st^, 2^nd^	NR	NR	NR	191	31	92	14	99	5 star	different detecting method, different therapy line, only Asian, not report PS
Shimizu T 2012 [26]	Asian	50	RT-PCR	III/IV	Pemetrexed-based	1^st^, pre-treated	NR	0-3	66.8	50	NR	NR	NR	NR	5 star	different detecting method, different therapy line, only Asian
Takezawa K 2011 [30]	Asian	24	IHC	IIIb/IV	Pemetrexed + cisplatin/Pemetrexed + carboplatin	1^st^	NR	0-1	66	24	NR	NR	NR	NR	6 star	different detecting method, different therapy, only Asian
Wang ZK 2010 [29]	Asian	38	RT-PCR	IIIb/IV	Pemetrexed + cisplatin	1^st^	≥2	0-2	48.6	38	11	28	2	10	6 star	different detecting method, different therapy, only Asian

Abbreviations: NR, no reported; IHC, immunohistochemistry; RT-PCR, real-time reverse transcriptase PCR

### Response Rate

**Figure 2:**
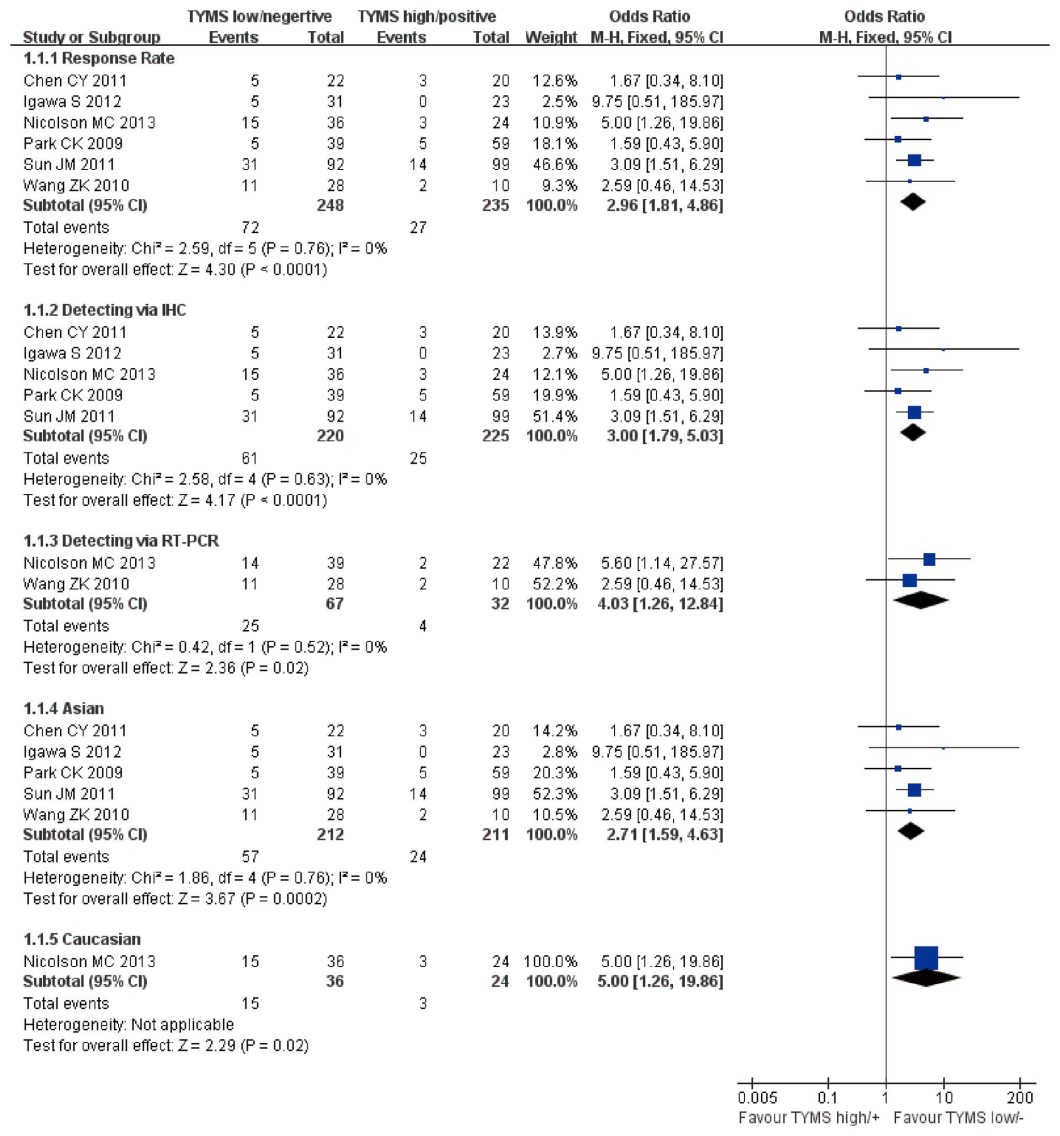
Fixed-effect model forest plot of Odds Ratio of response to pemetrexed-based regimen: TYMS low/negative vs. TYMS high/positive. The pooled OR of response rate is symbolized by a solid diamond at the bottom of the forest plot and the width of which represents the 95% CI.

**Figure 3:**
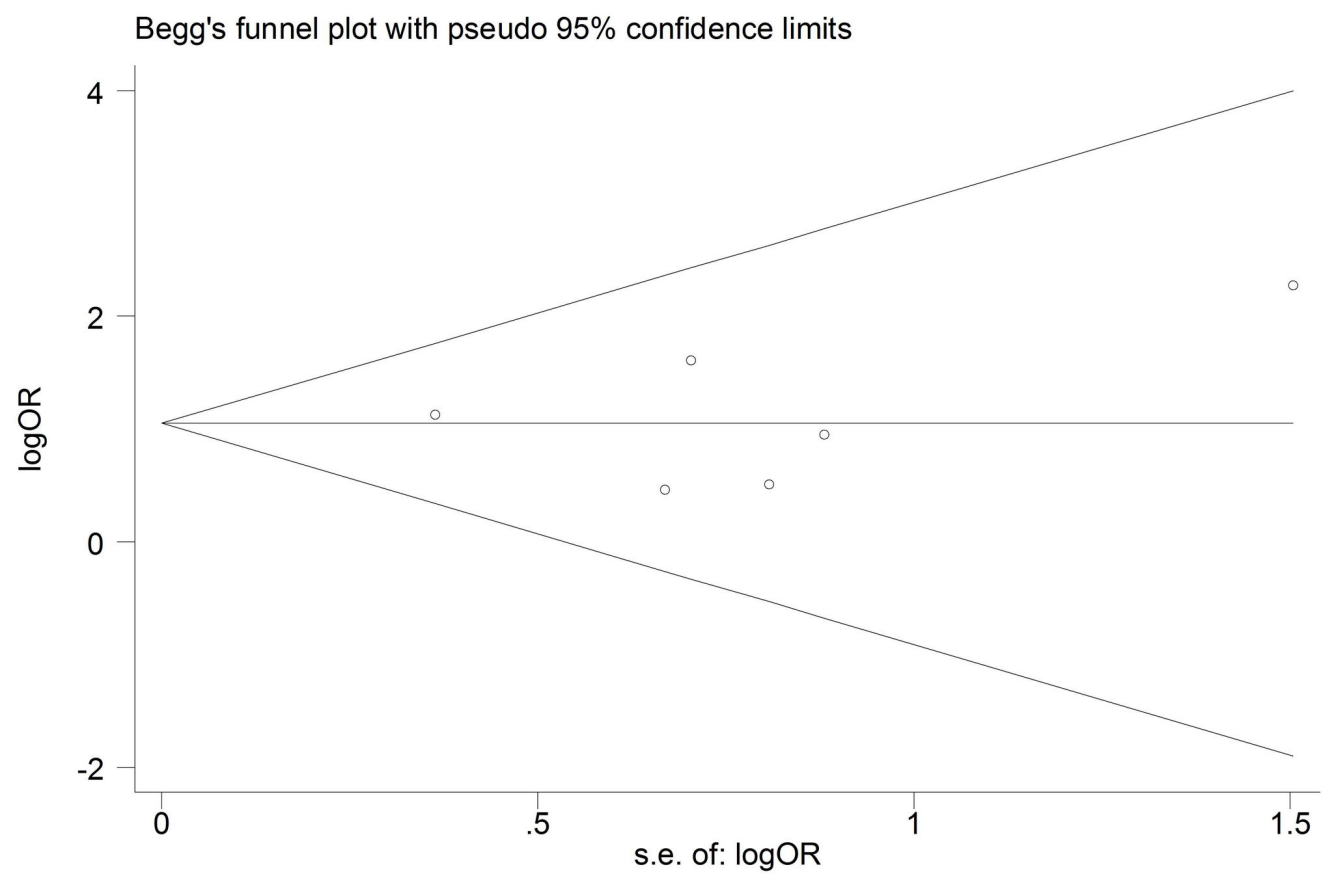
Funnel plot for publication bias test RR. The two oblique lines indicate the pseudo 95% confidence limits.

Six studies (n=483) compared the objective response between TYMS low/negative group with TYMS high/positive group [21–23,29,32,33]. No heterogeneity was found among studies (Chi^2^=2.59, *P*=0.76, I^2^= 0%). We used fixed effect model to perform meta-analysis. Pooled data showed that the overall objective response rate was significantly higher in TYMS low/negative expression group (OR=2.96, 95%CI [1.81, 4.86] *P*<0.0001; Figure 2). That showed the low/negative expression of TYMS was associated with higher response rate to pemetrexed-based chemotherapy. No evidence of publication bias was found in the funnel plots and Begg’s test (*P*=0.707; Figure 3).

The subgroup analysis was performed according to the detecting methods and ethnicity. IHC was used to detect TYMS in five studies and RT-PCR was used in the two studies. There was no heterogeneity among studies in IHC and RT-PCR subgroups (Chi^2^=2.58, *P*= 0.63, I^2^=0%; Chi^2^=0.42, *P*= 0.52, I^2^=0%). Fixed-effect model was used to perform subgroup meta-analysis. In the IHC and RT-PCR subgroups there were significant correlation between low/negative expression of TYMS and higher response rate to pemetrexed-based chemotherapy (OR=3.00, 95%CI [1.79, 5.03] *P*<0.0001; OR=4.03, 95%CI [1.26, 12.84] *P*=0.02; Figure 2).

There were five studies performed in Asians and one in Caucasians. No heterogeneity among studies were found in Asian subgroup (Chi^2^=1.86, *P*= 0.76, I^2^=0%). Fixed-effect model was used to perform subgroup analysis. A significant association between low/negative expression of TYMS and higher response rate was found in both Asian and Caucasian subgroups (OR=2.71, 95%CI [1.59, 4.63] *P*=0.0002; OR=5.0, 95%CI [1.26, 19.86] *P*=0.02; Figure 2).

### Progression Free Survival

**Figure 4:**
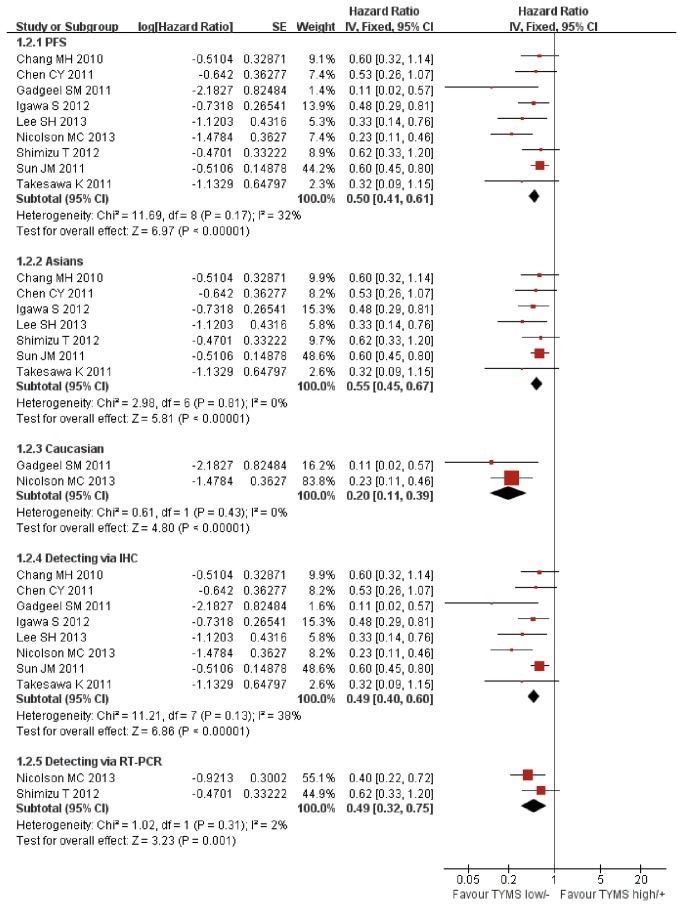
Fixed-effect model forest plot of Hazard Ratio of progression free survival according to the expression of TYMS: TYMS low/negative vs. TYMS high/positive. The pooled HR of PFS is symbolized by a solid diamond at the bottom of the forest plot and the width of which represents the 95% CI.

**Figure 5:**
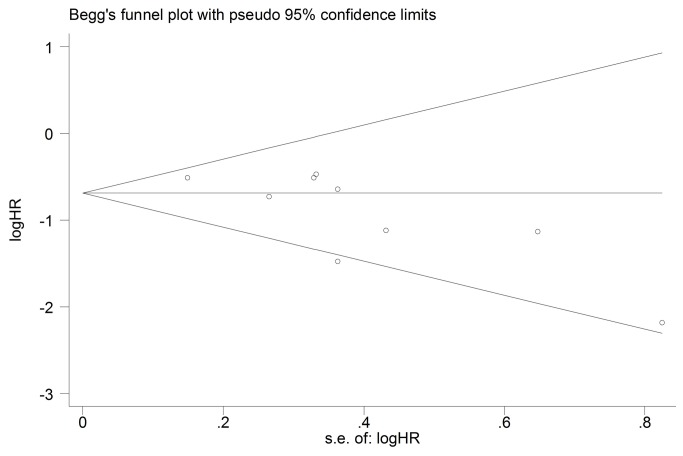
Funnel plot for publication bias test PFS. The two oblique lines indicate the pseudo 95% confidence limits.

Progression free survival data were available in 9 studies (*n*=662) [21–26,30–32]. No significant heterogeneity was found (Chi^2^=11.69, *P*=0.17, I^2^=32%). We used fixed effect model to perform meta-analysis. Pooled analysis showed that low/negative expression of TYMS was associated with a significant progression free survival benefit in advanced NSCLC patients treated with pemetrexed-based chemotherapy (HR 0.50, 95%CI [0.41, 0.61] *P*<0.00001; Figure 4). No evidence of publication bias was found in the funnel plots and Begg’s test (p=0.076; Figure 5).

The subgroup analysis was performed according to the detecting methods and ethnicity. There were 7 studies performed in Asians and 2 studies performed in Caucasians. Though there was no evidence of significant heterogeneity in both Asian and Caucasian subgroups (Chi^2^=2.98, *P*=0.81, I^2^= 0%; Chi^2^=0.61, *P*=0.43, I^2^= 0%), we used fixed-effect model to perform the analysis. In the subgroup analysis pooled data showed a significant association between low/negative expression of TYMS with longer progression free survival in Asian patients (HR 0.55, 95%CI [0.45, 0.67] *P*<0.00001; Figure 4). In Caucasian patients, the association between low/negative expression of TYMS with longer progression free survival was also significant (HR 0.20, 95%CI [0.11, 0.39] *P*<0.00001; Figure 4).

Eight studies used the IHC to detect the TYMS expression and only two studies used RT-PCR. No significant heterogeneity was found in the IHC and RP–PCR subgroups (Chi^2^=11.21, *P*=0.13, I^2^=38%; Chi^2^=1.02, *P*=0.31, I^2^=2%). The fixed-effect model was used too perform the meta-analysis in two subgroups. Subgroup analysis based on detecting method showed that low/negative expression of TYMS detected by IHC was significantly associated with longer progression free survival in advanced NSCLC patients treated with pemetrexed-based chemotherapy (HR 0.49, 95%CI [0.40, 0.60] *P*<0.00001; Figure 4). Such significance was also found in the RT-PCR subgroup (HR 0.49, 95%CI [0.32, 0.75] *P*=0.001; Figure 4).

### Overall Survival

**Figure 6:**
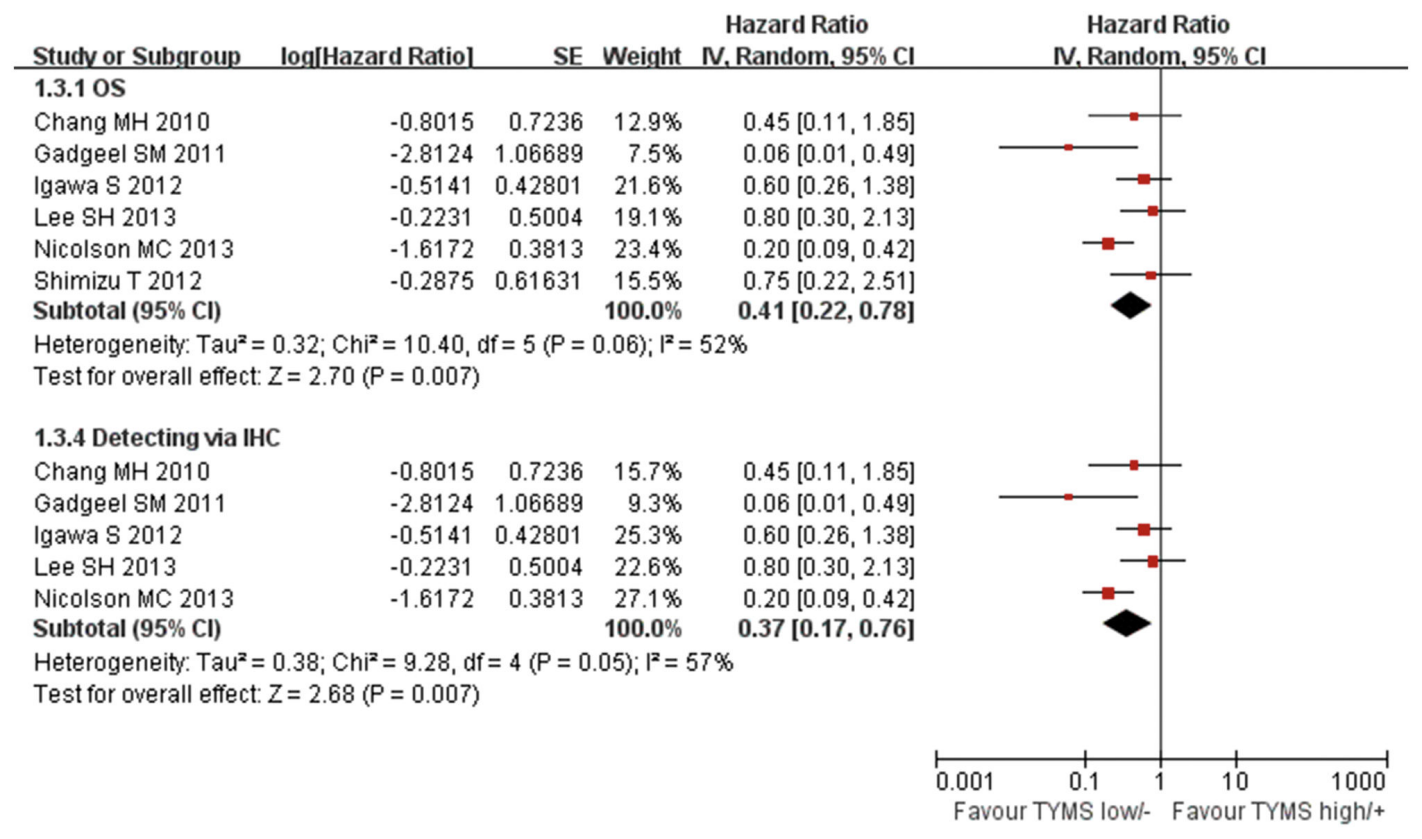
Randomized-effect model forest plot of Hazard Ratio of overall survival and in IHC subgroup analysis according to the expression of TYMS: TYMS low/negative vs. TYMS high/positive. The pooled HR of OS is symbolized by a solid diamond at the bottom of the forest plot and the width of which represents the 95% CI.

**Figure 7:**
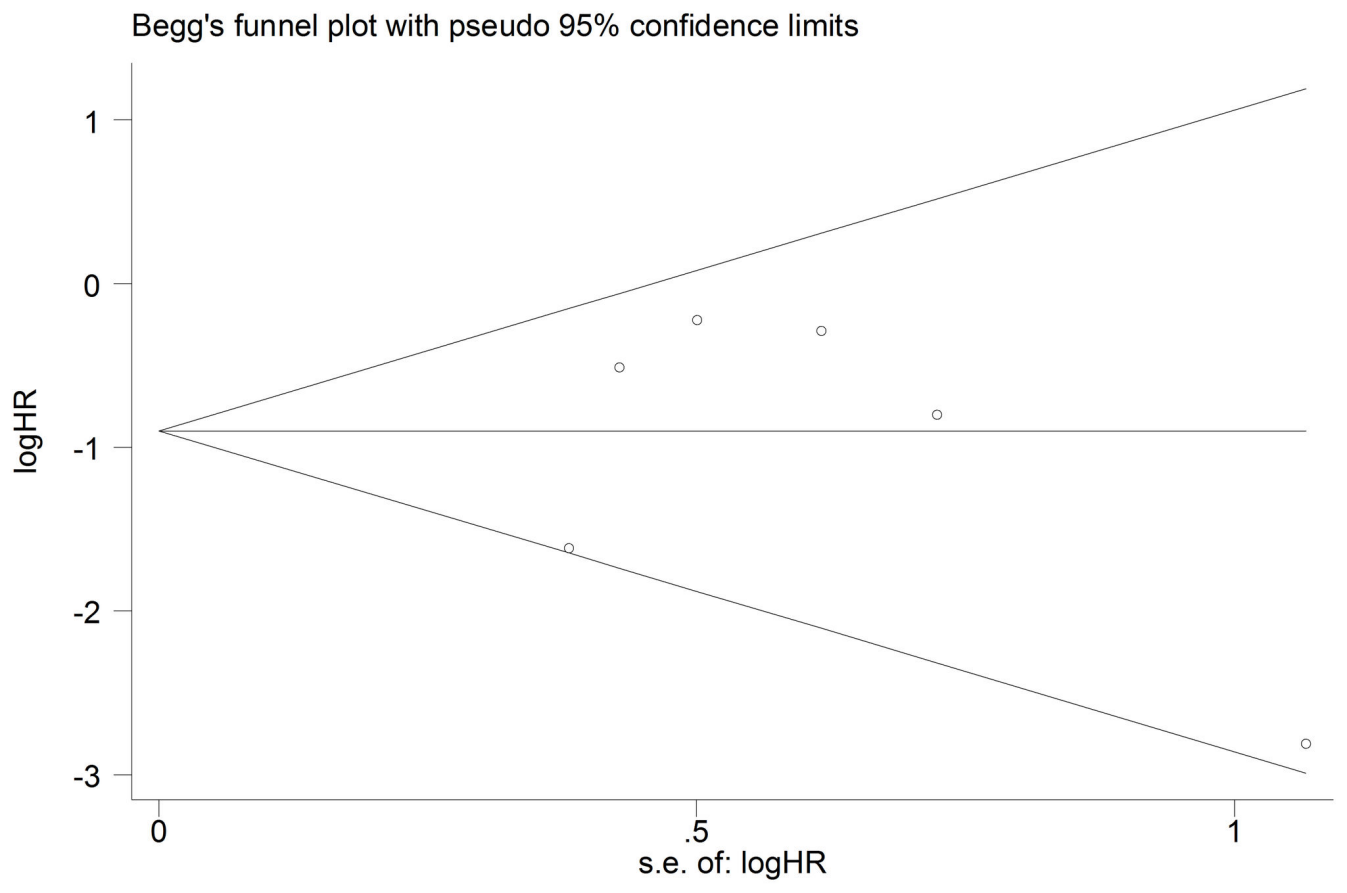
Funnel plot for publication bias test OS. The two oblique lines indicate the pseudo 95% confidence limits.

Eight studies compared the median survival time, but only 6 studies reported sufficiency data to carry out the meta-analysis [23–26,31,32]. Significant heterogeneity was found among studies (Chi^2^=10.4, *P*=0.06, I^2^= 52%). We used random effect model to perform meta-analysis. The pooled data showed that low/negative expression of TYMS was associated with a significant median survival advantage in advanced NSCLC patients receiving pemetrexed-based regimen(HR 0.41, 95%CI [0.22, 0.78] *P*=0.007; Figure 6). No evidence of publication bias was found in the funnel plots and Begg’s test (*P*=1.0; Figure 7).

**Figure 8:**
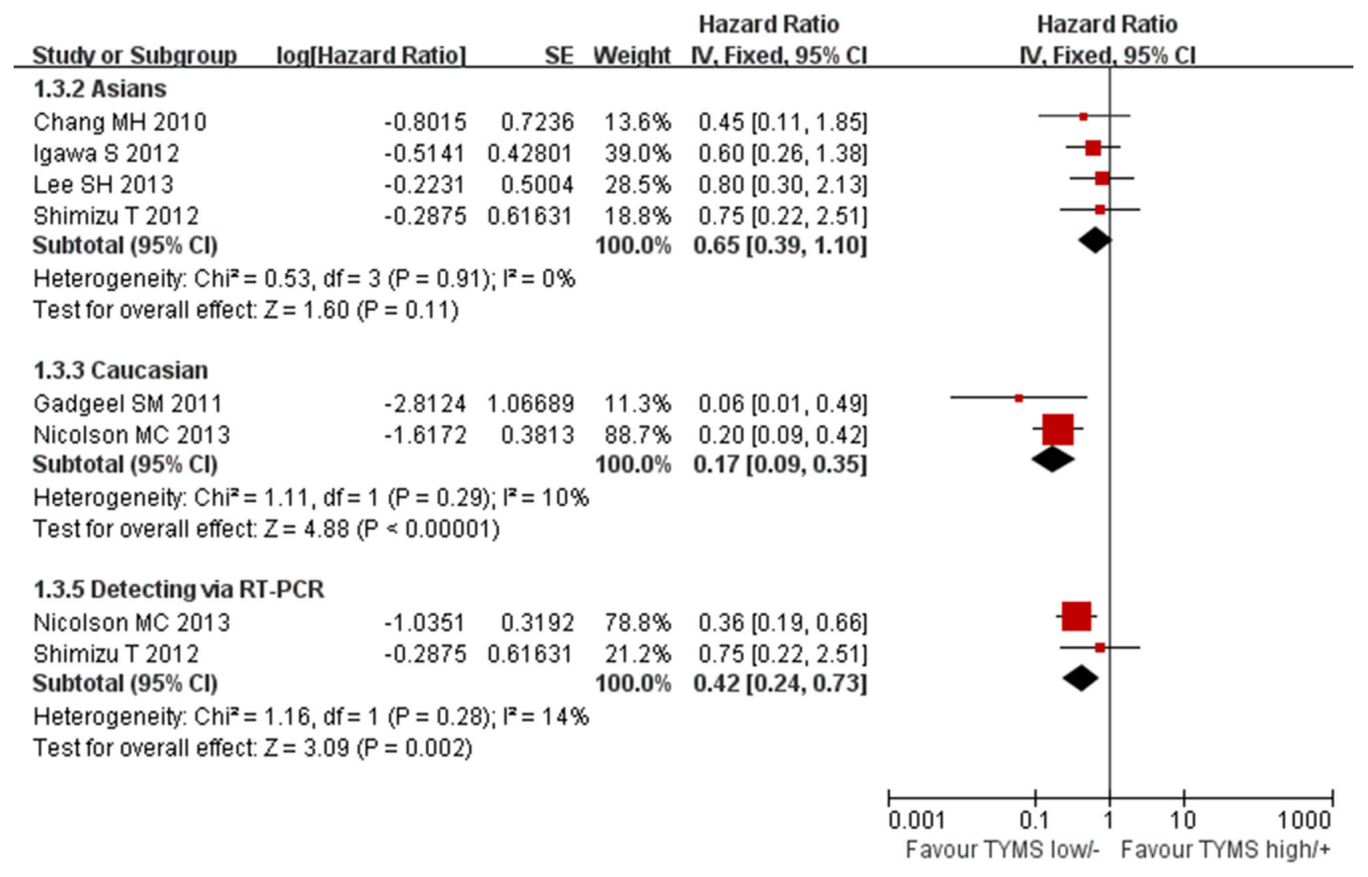
Fixed-effect model forest plot of Hazard Ratio of overall survival in Asian, Caucasian and RT-PCR subgroup analysis according to the expression of TYMS: TYMS low/negative vs. TYMS high/positive. The pooled HR of OS is symbolized by a solid diamond at the bottom of the forest plot and the width of which represents the 95% CI.

The subgroup analysis was performed according to the detecting methods and ethnicity. Four studies were performed in Asians and two in Caucasians. No heterogeneity was found in the Asian and Caucasian subgroups (Chi^2^=0.53, *P*=0.91, I^2^=0%; Chi^2^=1.11, *P*=0.29, I^2^=10%; Figure 8). The fixed-effect model was used in the subgroup analysis. We didn’t found significant association between low/negative expression of TYMS with longer overall survival in Asian subgroup (HR 0.65, 95%CI [0.39, 1.10] *P*=0.11; Figure 8). But in Caucasian subgroup, low/negative expression of TYMS was significantly associated with longer overall survival (HR 0.17, 95%CI [0.09, 0.35] *P*<0.00001; Figure 8).

Five studies used the IHC to detect the TYMS expression and two studies used RT-PCR. Significant heterogeneity was found in the IHC subgroup but not in RT-PCR subgroup (Chi^2^=9.28, *P*=0.05, I^2^=57%; Figure 6; Chi^2^=1.16, *P*=0.28, I^2^=14%; Figure 8). We used the random-effect model in IHC subgroup analysis and fixed-effect model in RT-PCR subgroup. Subgroup analysis based on detecting method showed that no matter detected by IHC or by RT-PCR low/negative expression of TYMS was significantly associated with longer overall survival (HR 0.37, 95%CI [0.17, 0.76] *P*=0.007; Figure 6; HR 0.42, 95%CI [0.24, 0.73] *P*=0.002; Figure 8).

## Discussion

The studies about relationship between TYMS expression and effect of pemetrexed-based chemotherapy were comparatively few, and reports about prognostic significance of TYMS expression in advanced NSCLC non-small cell lung cancer are controversial. So it is necessary to combine and analyze these data to find a result. Our purpose was to prove the hypothesis that low TYMS expression is associated with higher response rate and longer survival in advanced NSCLC non-small cell lung cancer patients. Our study may provide a theoretical evidence for individualized chemotherapy in advanced NSCLC and supports the use of detecting lung cancer tissue for TYMS expression to help us chose chemotherapy regimens.

To our knowledge there is no published meta-analysis about the predictive value of TYMS expression for pemetrexed-based chemotherapy in NSCLC patients. In this meta-analysis 11 studies were included, and they were most retrospective studies. TYMS expression was detected by immunohistochemistry and RT-PCR. Six of included studies compared the response rate between two groups. Nine studies reported the PFS and were included in analysis. Eight studies compared the OS but only six studies reported enough data to carry out the analysis. No study reported treatment related adverse effect between groups. This meta-analysis included a total of 798 cases of patients and demonstrated that the response rate was significantly higher and median survival (PFS and OS) were significantly longer in patients with low/negative TYMS expression.

Most of the included studies used IHC to detest the TYMS expression, while only two studies used RT-PCR. IHC and RT-PCR detects TYMS expression at protein level and mRNA level respectively. We all know that the expression of TYMS protein may influenced by many factors during several step, such as transcription, post-transcriptional regulation, translation and post-translation modification. The mRNA expression may be quite different from protein expression. In our meta-analysis one study published by Shimizu T compared the IHC and RT-PCR method in the detection effect of TYMS expression and reported that there was a significant correlation between the two detection methods [26]. However, most studies utilized IHC to detect the TYMS expression. Our analysis showed that TYMS expression detected by both IHC and RT-PCR was associated with higher response rate, longer PFS and longer OS. According to the results and available evidence, IHC is more preferable than RT-PCR when used to predict the sensitivity of pemetrexed-based regimens in patients with advanced NSCLC. Furthermore, the TYMS staining within the tumors varies a lot among studies and there’s lack of a standardized scoring system in NSCLC. These reasons may contribute to the heterogeneity. The reported TYMS positivity rate ranges from 29.6% to 72.5% [34–39].

This study has several other limitations. Heterogeneity is a potential problem to affect the results. We didn’t observed significant heterogeneity among studies in the analysis of response rate and PFS, but in OS analysis and subgroup analysis significant heterogeneity was observed among the studies of IHC subgroup. Many factors might cause significant heterogeneity, such as different stage, previous treatments, pathological subtype, treatment regimens, treatment cycles and performance status. Most study included the patients with stage III/IV while one study included relapsed patients. The relapsed patients may have longer survival than the advanced patients. The concurrent treatment regimens (radiotherapy, chemotherapy) and previous treatment (surgery, radiotherapy or chemotherapy) will influence the response rate and survival outcome a lot. What’s more, the combined treatment, especially with radiotherapy, will be more attend to achieve better response rate than single agent pemetrexed therapy.

Besides, another contributing factor might be ethnic differences, which may also affect the result. Most included studies were from Asia, and two studies were performed in Caucasian (Table 1). So, based on data from two retrospective studies, the result was not very representative and convincing in Caucasian patients until more evidence exists. Publication bias is also a possible limitation. However, in our study we didn’t find significant publication bias that might influence the result of meta-analysis.

In conclusion, despite the limitations of this meta-analysis, our study still demonstrated that low/negative TYMS expression was significantly associated with higher response rate, longer median overall survival and longer progression free survival for advanced NSCLC patients receiving pemtrexed-containing chemotherapy. Hence, TYMS may be a potential predictor of sensitivity to pemtrexed-based chemotherapy in advanced NSCLC. However, nearly all of the available information regarding the predictive value of TYMS was derived from retrospective studies. Large scale prospective clinical trials are still warranted to validate the prospective utility of TYMS in clinical decision making and appropriate marker evaluation methodology.

## Supporting Information

Checklist S1 **PRISMA Checklist.**
(DOC)
