# Supplementary material for: Association between TYMS Expression and Efficacy of Pemetrexed–Based Chemotherapy in Advanced Non-Small Cell Lung Cancer: A Meta-Analysis
Source: PLoS One. 2013 Sep 10;8(9):e74284. doi: 10.1371/journal.pone.0074284 (PMC3769376; doi:10.1371/journal.pone.0074284)
Supplement: Checklist S1 — PRISMA Checklist. (DOC) [file pone.0074284.s001.doc]

| **Section/topic** | **#** | **Checklist item** | **Reported on page #** |
| --- | --- | --- | --- |
| **TITLE** | | |  |
| Title | 1 | Association between TYMS expression and efficacy of pemetrexed–based chemotherapy in advanced non-small cell lung cancer: A meta-analysis | Title |
| **ABSTRACT** | | |  |
| Structured summary | 2 | Background: The predictive value of thymidylate synthase (TYMS) to sensitivity to pemetrexed-based chemotherapy in advanced non-small cell lung cancer (NSCLC) patients is controversial. We conducted a meta-analysis of all relevant published data to assess the association of TYMS expression with the clinical outcomes of pemetrexed-based regimen in advanced NSCLC.  Patients and methods: We conducted an electronic search using PubMed, and CNKI databases. Pooled odds ratio (OR) for the response rate and hazard ratio (HR) for the overall survival and progression free survival were calculated using the software Revman 5.0.  Results: There were 9 studies (n=663) met our criteria for evaluation. Response rate to pemetrexed-based regimen was significantly higher in patients with low/negative TYMS (OR=0.30, 95%CI [1.66, 5.41], P=0.0003). Patients with low/negative TYMS who were treated with pemetrexed-based regimen had longer overall survival (HR 0.54, 95%CI [0.38, 0.77], P=0.0006) and progression free survival (HR 0.57, 95%CI [0.48, 0.68], P<0.00001) than those with high/positive TYMS.  Conclusions: Low/negative TYMS expression was significantly associated with higher response rate, longer median survival and longer progression free survival for advanced NSCLC patients receiving pemtrexed-based chemotherapy. Hence, TYMS may be an important predictor of sensitivity to pemtrexed-based chemotherapy in advanced NSCLC. | Abstract |
| **INTRODUCTION** | | |  |
| Rationale | 3 | Lung cancer has been estimated as the most common cancer in the world for several decades. Pemetrexed combined with platinum or alone is approved as standard first or second-line treatment for advanced NSCLC. thymidylate synthase (TYMS) is one of the pharmacologic targets of pemetrexed. Several recent studies reported that low TYMS expression was associated with better response and/or survival when treated with pemtrexed-based regimens in NSCLC patients. But some other studies didn’t show the significant association between TYMS expression and efficacy of pemtredxed-based chemotherapy in NSCLC. However, the association between TYMS expression and treatment efficacy of pemetrexed in NSCLC is unclear. | Introduction |
| Objectives | 4 | The main objective of this study is to evaluate whether thymidylate synthase (TYMS) is a predictive biomarker of efficacy of pemetrexed-based regimen in advanced NSCLC. | Introduction |
| **METHODS** | | |  |
| Protocol and registration | 5 | This research use the PRISMA (Preferred Reporting Items for Systematic Reviews and Meta-Analyses) statement as a guide.(available at：http://www.prisma-statement.org) | Methods and Patients |
| Eligibility criteria | 6 | Case-control and cohort studies, published in English only. | Methods and Patients |
| Information sources | 7 | Studies were searched in databases PubMed and CNKI from inception to May 2013. | Methods and Patients |
| Search | 8 | Studies were searched using the search terms of (thymidylate synthase OR TYMS OR TS) and (non-small cell lung cancer OR non-small cell lung carcinoma) and pemetrexed and chemotherapy, and any combination of key words were used to electronic search. The manual search was applied in the reference of included studies. We only searched the articles published in English. | Methods and Patients |
| Study selection | 9 | Studies that met the following criteria were included: (1) Studies included should be published, regardless of its design, publication language or date. (2)The study subjects should be patients with pathologically proven advanced NSCLC under pemetrxed-containing regimens. (3) TYMS expression should be detected with immunohistochemistry (IHC) or real-time reverse transcriptase PCR (RT-PCR).  Studies that didn’t provide at least one outcome of objective response rate, median survival or survival time will be excluded. | Methods and Patients |
| Data collection process | 10 | Data extraction was conducted independently by 2 investigators and Quality of the studies was assessed using the Newcastle-Ottawa Quality Assessment Scale for case-control and cohort studies by 2 independent evaluators. Data were adjudicated by 2 additional investigators according to the original articles after data extraction and assessment. Any disagreement will be present to discuss within all authors. | Methods and Patients |
| Data items | 11 | Author name, publication time, ethnicity, study design, sample size, age, disease stage, ECOG PS, TYMS detection method and outcomes (response rate, overall survival, progression free survival and hazard ratio of survival data). If original hazard ratio was not reported the Kaplan–Meier curves will be used to extract hazard ratio estimation according to the methods described by Tierney in 2007. | Methods and Patients |
| Risk of bias in individual studies | 12 | The report quality of studies was assessed using the Newcastle-Ottawa Quality Assessment Scale for case-control and cohort studies, including patient selection, study comparability and outcome by 2 independent evaluators. This scale is an eight-item instrument that allows for assessment of patient population and selection, study comparability, follow-up, and outcome of interest. Interpretation of the scale is performed by awarding points, or stars’, for high-quality elements. Stars are then added up and used to compare study quality in a quantitative manner. | Methods and Patients |
| Summary measures | 13 | Odds Ratio, Hazard Ratio | Methods and Patients |
| Synthesis of results | 14 | The association between TYMS and response rate was expressed as odds ratio (OR). The association between TYMS and PFS or OS was expressed as a hazard ratio (HR).  Statistical heterogeneity between studies was examined using both the Cochrane Q statistic (significant at P<0.1) and the I^2^ value. I^2^ >50% were considered to represent significant heterogeneity respectively. A fixed-effect model was used when heterogeneity was not detected by chi-square test (P>0.10); otherwise, a random-effect model was used. All statistical analysis was performed by Review manager 5.0 (http://www.cochrane.org). The pooled OR and its 95% confidence intervals (CIs) were calculated using Mantel–Haenszel formula (fixed-effect model) or Dersimonian–Laird formula (random-effect model). For quantitative evaluation of PFS and OS results, HR was used to estimate the impact of TYMS expression on PFS and OS of patients received pemetrexed-based chemotherapy. HR, its variance, its 95% CI, log(HR) and se(log(HR)) for each study were extracted or calculated based on the published researches according to the methods described by Tierney [28]. Kaplan-Meier curves were read by Engauge Digitizer version 4.1 (http://digitizer.sourceforge.net/). A significant two-way P value for comparison was defined as P<0.05. The results were described by forest plots, every square represents each study’s OR or HR estimate. The pooled OR or HR is symbolized by a solid diamond at the bottom of the forest plot and the width of the square represents the 95% CI of OR or HR. The size of the square represents the weight that the corresponding study exerts in the meta-analysis. | Methods and Patients |

Page 1 of 2

| **Section/topic** | **#** | **Checklist item** | **Reported on page #** |
| --- | --- | --- | --- |
| Risk of bias across studies | 15 | The potential publication bias was evaluated by funnel plots. | Methods and Patients |
| Additional analyses | 16 | Subgroup analysis was performed to explore the influence of ethnicity and detecting method in the outcomes. | Methods and Patients |
| **RESULTS** | | |  |
| Study selection | 17 | Fig 1. Flow chart of the search result of the meta-analysis. | Results |
| Study characteristics | 18 | | Study(year) | Ethnicity | Patient  (N) | TYMS  detecting  method | Disease  stage | ECOG  PS | Age  (year) | Evaluable  For  response | TYMS  low/negative | | TYMS  high/positive | | Quality score | | --- | --- | --- | --- | --- | --- | --- | --- | --- | --- | --- | --- | --- | |  | (n) | RR(n) | Total(n) | RR(n) | Total(n) | | Chang MH 2010 | Asian | 110 | IHC | IIIb/IV | 0-3 | 59 | 52 | NR | NR | NR | NR | 6 star | | Chen CY 2011 | Asian | 42 | IHC | IIIb/IV | NR | 61.5 | 42 | 5 | 22 | 3 | 20 | 6 star | | Christoph DC 2013 | Caucasian | 207 | IHC | IV | NR | 59 | 196 | NR | NR | NR | NR | 7 star | | Igawa S 2012 | Asian | 104 | IHC | IIIb/IV | 0-3 | 65 | 54 | 5 | 31 | 0 | 23 | 6 star | | Gadgeel SM 2011 | Caucasian | 28 | IHC | IIIa/IIIb | 0-1 | 60 | 16 | NR | NR | NR | NR | 6 star | | Sun JM 2011 | Asian | 193 | IHC | IIIb/IV | NR | NR | 191 | 31 | 92 | 14 | 99 | 6 star | | Shimizu T 2012 | Asian | 50 | RT-PCR | III/IV | 0-3 | 66.8 | 50 | NR | NR | NR | NR | 5 star | | Takezawa K 2011 | Asian | 24 | IHC | IIIb/IV | NR | 66 | 24 | NR | NR | NR | NR | 5 star | | Wang ZK 2010 | Asian | 38 | RT-PCR | IIIb/IV | 0-2 | 48.6 | 38 | 11 | 28 | 2 | 10 | 5 star |  | 21. Chen CY, C.Y., Shih JY, Lin JW, Chen KY, Yang CH, Yu CJ, Yang PC, Chang YL, Shih JY, Lin JW, Chen KY, Yang CH, Yu CJ,Yang PC, *Thymidylate synthase and dihydrofolate reductase expression in non-small cell lung carcinoma: the association with treatment efficacy of pemetrexed.* Lung Cancer, 2011. 74(1): p. 132-8.  22. Sun JM, H.J., Ahn JS, Park K, Ahn MJ., *Significance of thymidylate synthase and thyroid transcription factor 1 expression in patients with nonsquamous non-small cell lung cancer treated with pemetrexed-based chemotherapy.* J Thorac Oncol, 2011. 6(8): p. 1392-9.  23. Igawa, S., et al., *Pemetrexed for Previously Treated Patients with Non-Small Cell Lung Cancer and Differences in Efficacy according to Thymidylate Synthase Expression.* Chemotherapy, 2012. 58(4): p. 313-320.  24. Gadgeel SM, R.J., Patel BB, Wozniak A, Konski A, Valdivieso M, Hackstock D, Chen W, Belzer K, Burger AM, Marquette L, Turrisi A, *Phase II Study of Pemetrexed and Cisplatin, with Chest Radiotherapy Followed by Docetaxel in Patients with Stage III Non-small Cell Lung Cancer.* J Thorac Oncol, 2011. 6(5): p. 927-33.  25. Chang MH, A.J., Lee J, Kim KH, Park YH, Han J, Ahn MJ, Park K., *The efficacy of pemetrexed as a third- or fourth-line therapy and the significance ofthymidylate synthase expression in patients with advanced non-small cell lung cancer.* Lung Cancer, 2010. Sep;69(3): p. 323-9.  29. Christoph DC, H.A., Gauler TC, Asuncion BR, Loewendick H, Peglow A, Hassan B, Tran C, Wynes MW, Schuler M, Eberhard WE, Hirsch FR, *Detection of circulating lung cancer cells with strong thymidylate synthase reactivity in the peripheral blood of a patient with pulmonary adenocarcinoma treated with pemetrexed.* J Thorac Oncol, 2012. 7(4): p. 766-7.  30. Shimizu T, N.Y., Nakagawa Y, Tsujino I, Takahashi N, Nemoto N, Hashimoto S., *Association between expression of thymidylate synthase, dihydrofolate reductase, and glycinamide ribonucleotide formyltransferase and efficacy of pemetrexed in advanced non-small cell lung cancer.* Anticancer Res, 2012. 32(10): p. 4589-96.  31. Wang ZK, et al., *[Thymidylate synthase expression and therapeutic effect analysis of pemetrexed in advanced lung adenocarcinoma].* Nan Fang Yi Ke Da Xue Xue Bao, 2010. 30(5): p. 978-80.  32. Takezawa K, O.I., Okamoto W, Takeda M, Sakai K, Tsukioka S, Kuwata K, Yamaguchi H, Nishio K, Nakagawa K., *Thymidylate synthase as a determinant of pemetrexed sensitivity in non-small cell lung cancer.* Br J Cancer, 2011. 104(10): p. 1594-601. |  | | --- | --- | | Results |
| Risk of bias within studies | 19 | | Study(year) | Quality score | Risk of bias | | --- | --- | --- | | Chang MH 2010 | 6 star | different detecting method, different therapy line, only Asian | | Chen CY 2011 | 6 star | different detecting method, different therapy line, only Asian, not report PS | | Christoph DC 2013 | 7 star | different detecting method, different therapy line, not report PS, only Caucasian, | | Igawa S 2012 | 6 star | different detecting method, different therapy line, only Asian | | Gadgeel SM 2011 | 6 star | different detecting method, different therapy line, only Caucasian | | Sun JM 2011 | 6 star | different detecting method, different therapy line, only Asian, not report PS | | Shimizu T 2012 | 5 star | different detecting method, different therapy line, only Asian | | Takezawa K 2011 | 5 star | different detecting method, different therapy line, only Asian, not report PS | | Wang ZK 2010 | 5 star | different detecting method, different therapy line, only Asian | | Abbreviations: NR, not report; IHC, immunohistochemistry; RT-PCR, real-time reverse transcriptase PCR. | | | | Citations see Item 18 | | | | Results |
| Results of individual studies | 20 | 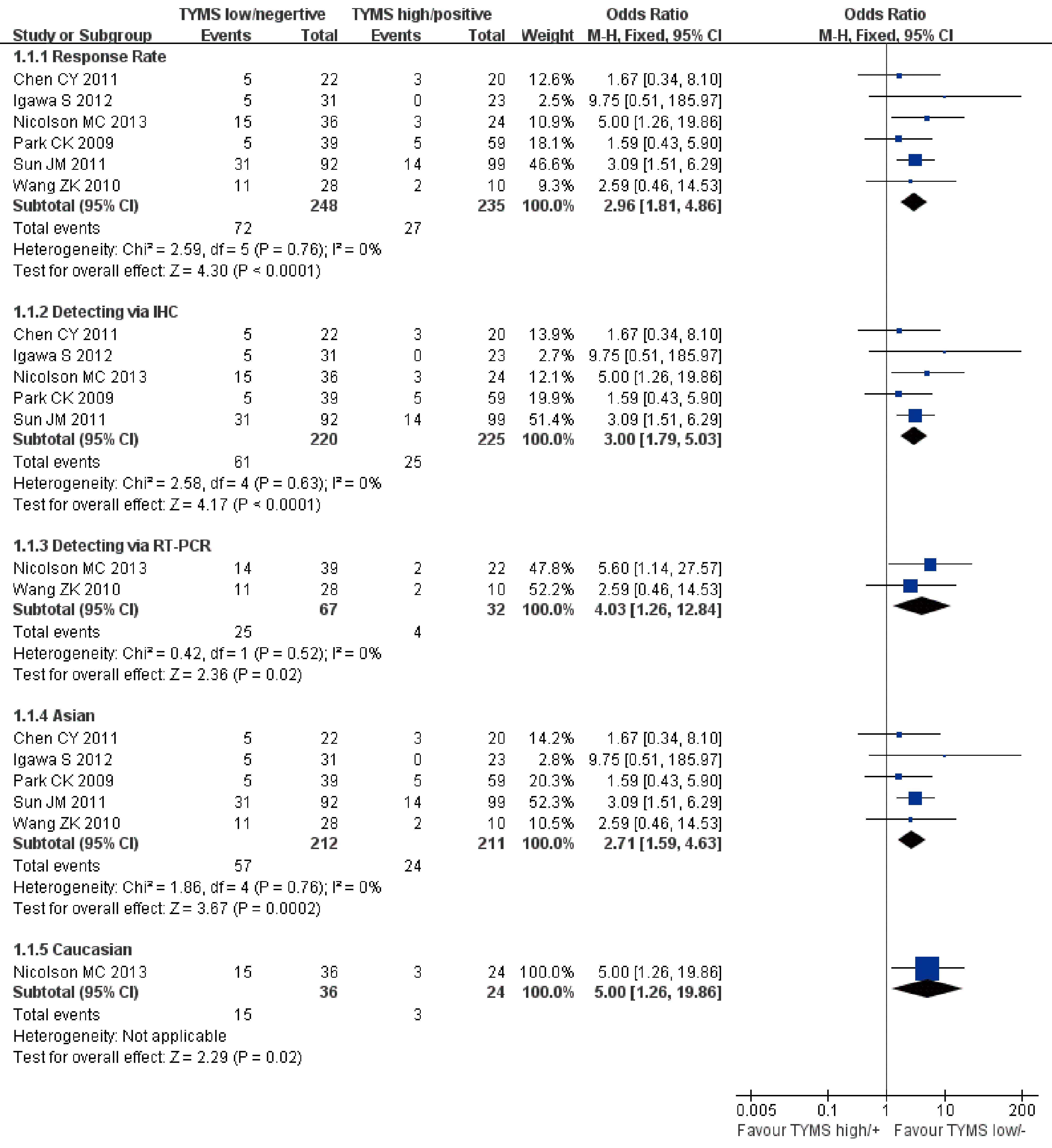  Figure 2. Fixed-effect model forest plot of Odds Ratio of response to pemetrexed-based regimen: TYMS low/negative vs. TYMS high/positive. The pooled OR of response rate is symbolized by a solid diamond at the bottom of the forest plot and the width of which represents the 95% CI.  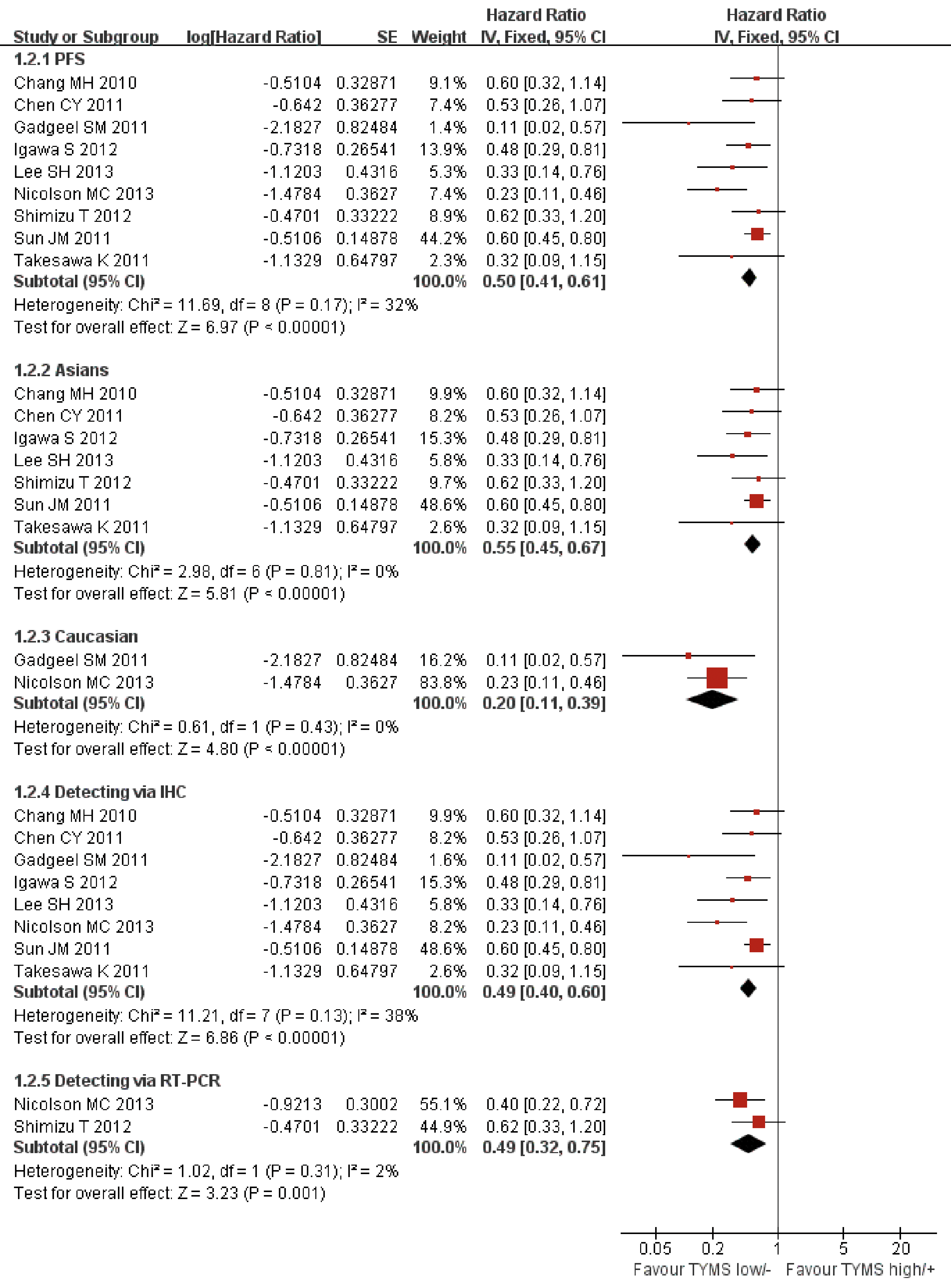  Figure 4. Fixed-effect model forest plot of Hazard Ratio of progression free survival according to the expression of TYMS: TYMS low/negative vs. TYMS high/positive. The pooled HR of PFS is symbolized by a solid diamond at the bottom of the forest plot and the width of which represents the 95% CI.  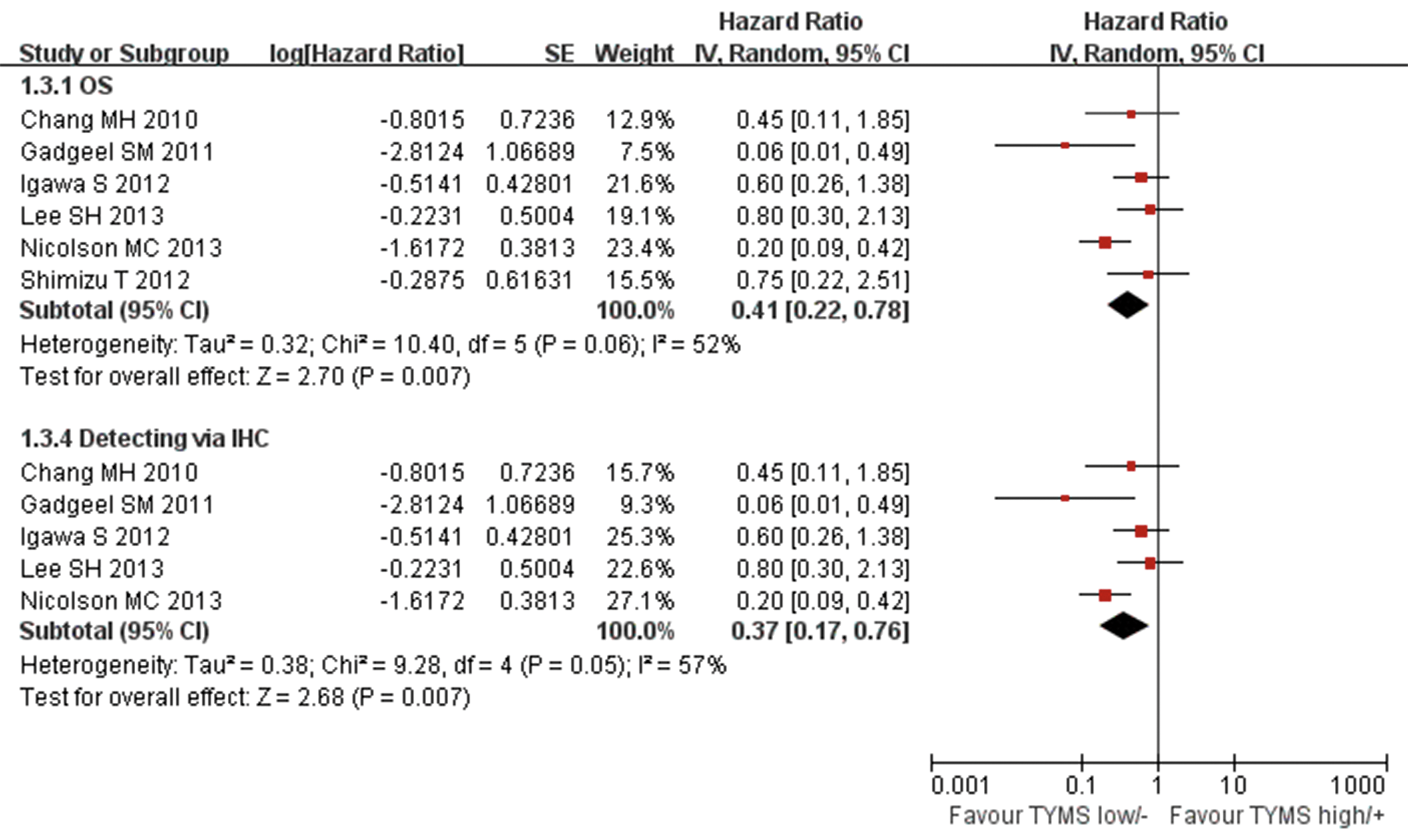  Figure 6. Randomized-effect model forest plot of Hazard Ratio of overall survival and in IHC subgroup analysis according to the expression of TYMS: TYMS low/negative vs. TYMS high/positive. The pooled HR of OS is symbolized by a solid diamond at the bottom of the forest plot and the width of which represents the 95% CI.  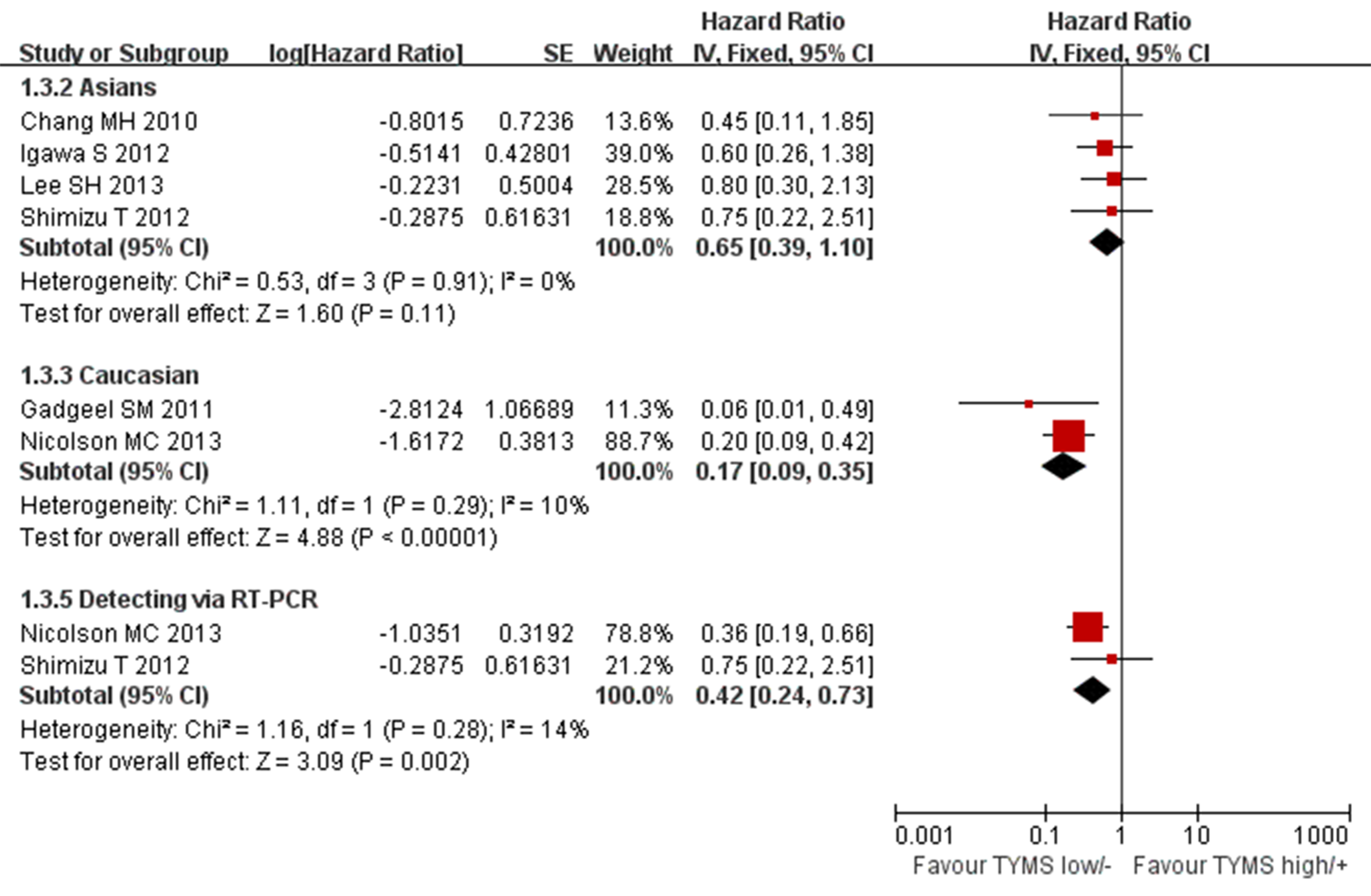  Figure 8. Fixed-effect model forest plot of Hazard Ratio of overall survival in Asian, Caucasian and RT-PCR subgroup analysis according to the expression of TYMS: TYMS low/negative vs. TYMS high/positive. The pooled HR of OS is symbolized by a solid diamond at the bottom of the forest plot and the width of which represents the 95% CI. | Results |
| Synthesis of results | 21 | **Response Rate**: OR=3.0, 95%CI [1.66, 5.41] P=0.0003 Fig 2. No heterogeneity was found among studies (Chi2=1.18, P=0.76, I2= 0%).  **Progression Free Survival**: HR 0.57, 95%CI [0.48, 0.68] Z=6.12 P<0.00001; Fig 3. No heterogeneity was found (Chi2=5.70, P=0.58, I2=0%).  **Overall Survival**: HR 0.54, 95%CI [0.38, 0.77] Z=3.41 P=0.0006; Fig 5. No heterogeneity was found among studies (Chi2=4.7, P=0.32, I2= 15%). | Results |
| Risk of bias across studies | 22 | Publication bias was not found according to funnel plots. | Results |
| Additional analysis | 23 | **Response Rate Subgroup Analysis**: In the IHC subgroup, OR=3.06, 95%CI [1.64, 5.73] P=0.0005; Fig 2. No heterogeneity was found among studies (Chi2=1.18, P=0.76, I2= 0%).  **Progression Free Survival Subgroup Analysis**:  I n Asian patients HR 0.57, 95%CI [0.46, 0.70] Z=5.34 P<0.00001; Fig 3. No evidence of heterogeneity in the Asian subgroup (Chi2=1.45, P=0.92, I2= 0%). In Caucasian patients HR 0.32, 95%CI [0.06, 1.70] Z=1.33 P=0.18; Fig 4. But in the Caucasian subgroup high heterogeneity was found (Chi2=4.21, P=0.04, I2=76%).  In IHC subgroup HR 0.57, 95%CI [0.47, 0.68] Z=5.96 P<0.00001; Fig 3. In RT-PCR subgroup HR 0.62, 95%CI [0.33, 1.20] Z=1.42 P=0.16; Fig 3. No heterogeneity was found (Chi2=5.62, P=0.47, I2=0%).  **Overall Survival Subgroup Analysis**: In Asian subgroup HR 0.60, 95%CI [0.32, 1.12] Z=1.16 P<0.11; Fig 5. In Caucasian subgroup HR 0.24, 95%CI [0.03, 2.02] Z=1.32 P=0.19; Fig 6. Though there was no evidence of heterogeneity in the Asian subgroup Chi2=0.29, P=0.86, I2=0%, but in Caucasian subgroup Chi2=4.25, P=0.04, I2=76%.  In IHC subgroup HR 0.53, 95%CI [0.37, 0.76] Z=3.43 P=0.0006; Fig 5. In RT-PCR subgroup HR 0.75, 95%CI [0.22, 2.51] Z=0.47 P=0.64 Fig 6. No heterogeneity was found in the IHC subgroup (Chi2=4.40, P=0.22, I2=32%). | Results |
| **DISCUSSION** | | |  |
| Summary of evidence | 24 | Our meta-analysis indicates that low/negative TYMS expression was significantly associated with higher response rate (OR=3.0, [1.66, 5.41] P=0.0003), longer progression free survival (HR 0.57, [0.48, 0.68] P<0.00001) and longer median overall survival (HR 0.54, [0.38, 0.77] P=0.0006) than high/positive TYMS expression for advanced NSCLC patients receiving pemtrexed-containing chemotherapy. | Discussion |
| Limitations | 25 | This study has several limitations. Heterogeneity is a potential problem to affect meta-analysis results. We didn’t observed significant heterogeneity among all studies, but in subgroup analysis significant heterogeneity was observed among the studies of Caucasians. The study result about PFS and OS reported by Gadgeel SM[30] were based on data from only 16 patients while the study by Christoph DC [29] included 207 patients. The notable difference of patient number between the two studies may cause the heterogeneity. Obviously the result of study included much more patients was more convincing.  Besides, many reasons, such as the detection method of TYMS, different evaluation standards of TYMS and different base-line characteristics, may affect the result. Publication bias is also a possible limitation. However, in our study we didn’t find significant publication bias that might influence the result of meta-analysis. | Discussion |
| Conclusions | 26 | In conclusion, despite the limitations of this meta-analysis, our study still demonstrated that negative/low TYMS expression was significantly associated with higher response rate, longer median overall survival and longer progression free survival for advanced NSCLC patients receiving pemtrexed-containing chemotherapy. Hence, TYMS may be an important predictor of sensitivity to pemtrexed-based chemotherapy in advanced NSCLC. However, nearly all of the available information regarding the predictive value of TYMS was derived from retrospective studies. Prospective clinical trials are still warranted to validate the prospective utility of TYMS in clinical decision making and appropriate marker evaluation methodology. | Discussion |
| **FUNDING** | | |  |
| Funding | 27 | Non | Non |

*From:*  Moher D, Liberati A, Tetzlaff J, Altman DG, The PRISMA Group (2009). Preferred Reporting Items for Systematic Reviews and Meta-Analyses: The PRISMA Statement. PLoS Med 6(6): e1000097. doi:10.1371/journal.pmed1000097

For more information, visit: **www.prisma-statement.org**.

Page 2 of 2
